# The Diagnostic Value of FibroTest and Hepascore as Non-Invasive Markers of Liver Fibrosis in Primary Sclerosing Cholangitis (PSC)

**DOI:** 10.3390/jcm12247552

**Published:** 2023-12-07

**Authors:** Bogdan Cylwik, Alicja Bauer, Ewa Gruszewska, Kacper Gan, Marcin Kazberuk, Lech Chrostek

**Affiliations:** 1 Department of Pediatric Laboratory Diagnostics, Medical University of Bialystok, 15-274 Bialystok, Poland; 2 Department of Biochemistry and Molecular Biology, Centre of Postgraduate Medical Education, 01-813 Warsaw, Poland; alabauer@wp.pl; 3 Department of Biochemical Diagnostics, Medical University of Bialystok, 15-269 Bialystok, Poland; ewa.gruszewska@umb.edu.pl (E.G.); lech.chrostek@umb.edu.pl (L.C.); 4 Department of Internal Diseases and Gastroenterology, Provincial Welded Hospital, 15-278 Bialystok, Poland; kacpergan@gmail.com (K.G.); marcin.kazberuk@gmail.com (M.K.)

**Keywords:** liver biopsy, non-invasive markers of liver fibrosis and cirrhosis, primary sclerosing cholangitis

## Abstract

The aim of this study was to evaluate the diagnostic usefulness of two non-invasive, validated, and patented markers of liver fibrosis, the Hepascore and FibroTest, in patients with primary sclerosing cholangitis (PSC). The study group consisted of 74 PSC patients and 38 healthy subjects. All patients had a liver biopsy. The Hepascore and FibroTest were calculated using specific algorithms. The ANOVA rank Kruskal–Wallis test revealed differences in the Hepascore and FibroTest between patients divided according to histological stage (*p* < 0.001 for both comparisons). The Hepascore and FibroTest had significantly higher results in patients with significant fibrosis (F ≥ 2) in comparison to those with no significant fibrosis (F1) (*p* < 0.001 for both tests) and higher values in patients with cirrhosis (F4) when compared to those without cirrhosis (F1–F3) (*p* < 0.001 for both comparisons). The Hepascore test showed a diagnostic sensitivity of 96.8%, a specificity of 100% for fibrosis (at cut-off 0.52) and a diagnostic sensitivity of 95.2%, and a specificity also of 100% for cirrhosis (at 0.80). The FibroTest in point 0.51 for the diagnosis of fibrosis obtained the following values: 58.6%, 90%, 89.5%, and 60%, respectively, and in point 0.73 for the diagnosis of cirrhosis: 42.9%, 100%, 100%, and 45.5, respectively. The Hepascore test reached an excellent diagnostic power in identifying both fibrosis and cirrhosis (AUC = 1.0). The FibroTest and Hepascore are highly valuable for the evaluation of the severity of liver fibrosis and cirrhosis in PSC patients and can be used as a primary screening method, allowing for a significant reduction in the need for liver biopsy. Both markers have the required sensitivity and specificity to detect liver fibrosis and cirrhosis and can be equally used in clinical practice, although the Hepascore seems to be a better test because it is more specific.

## 1. Introduction

Primary sclerosing cholangitis (PSC) is a chronic, progressive, hepatobiliary disease characterized by the inflammation and fibrosis of the intra- and extra-hepatic bile ducts, which over time leads to cirrhosis and liver failure [1,2]. The incidence and prevalence rates for PSC range from 0–1.3 and 0–16.2 per 1,000,000 persons in the population per year, respectively [3]. The mean age at diagnosis is between 30 and 40 years, and the male-to-female ratio is 2:1 [4]. The pathogenesis comprises a combination of genetic, environmental, infectious, and autoimmune factors. As many patients with PSC are asymptomatic with no clinical presentation, the diagnosis is often made incidentally by laboratory tests, and the most common laboratory abnormality is the elevation of serum alkaline phosphatase (ALP) and γ-glutamyltransferase (GGT) activity as markers of cholestasis [2]. In addition to laboratory tests, an imaging examination, and, in some cases, an endoscopy and liver biopsy are performed. The most important imaging test, constituting the gold diagnostic standard, is a magnetic resonance cholangiopancreatography (MRCP), which allows for the non-invasive imaging of the bile ducts (characteristic strictures and dilatations). In the case of an unclear picture from the MRCP or the collection of material from the bile ducts, an invasive endoscopic retrograde cholangiopancreatography (ERCP) examination is performed. When both examinations fail to confirm the diagnosis and the clinical picture indicates PSC, a liver biopsy and histopathological study of the specimen are needed. A liver biopsy is not necessary for all patients with PSC, but it is needed to diagnose two special subsets of patients with PSC: those with small duct PSC and those who show an overlap with autoimmune hepatitis [5,6].

During the course of PSC, the estimation of liver fibrosis progression to cirrhosis is very important. A quick evaluation of the severity of liver fibrosis is needed for prognosis and predicting potential complications in patients with PSC. Traditionally, liver biopsy has been considered the gold standard for the evaluation of the stage of liver fibrosis, but this is an invasive procedure and can lead to many complications. Therefore, new non-invasive techniques and methods are needed, including a laboratory test for the diagnosis of liver fibrosis and cirrhosis [7,8,9,10,11]. These tests can reflect fibrogenesis either indirectly or directly. Several non-invasive laboratory tests have already been used to assess liver fibrosis, including the APRI (Aspartate aminotransferase to Platelet Ratio Index) and the Fib-4 (Fibrosis-4) score of PSC [7,12]. The introduction of these biomarkers may reduce the need for liver biopsy and allow for the earlier treatment of PSC patients. Thus, the aim of this study was to evaluate the diagnostic usefulness of two non-invasive, validated, and patented markers of liver fibrosis, the Hepascore and FibroTest, in patients with PSC.

## 2. Materials and Methods

### 2.1. Patients

**Figure 1:**
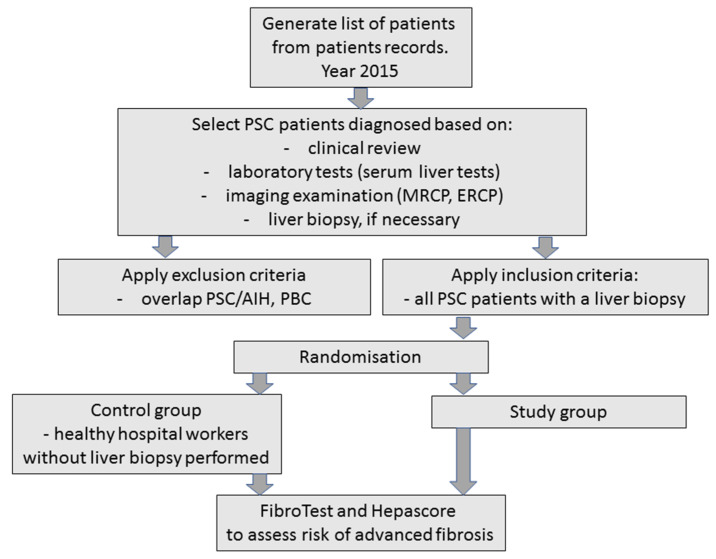
The flow chart of the recruitment and randomization of the patients in the study.

The study group consisted of 74 PSC patients (27 women, 47 men; median age: 35.5 years, range of 26–75 years) who were hospitalized in the Department of Gastroenterology, Hepatology and Clinical Oncology at the Center of Postgraduate Medical Education (Warsaw, Poland). The diagnosis of primary sclerosing cholangitis was based on the patient’s history, clinical examination, laboratory tests, and imaging investigations: magnetic resonance cholangiopancreatography (MRCP) or endoscopic retrograde cholangiopancreatography (ERCP) [6]. All patients had a liver biopsy taken and were divided according to the Ludwig’s score system for the severity of liver fibrosis into the following stages: F0, no fibrosis; F1, portal fibrosis without septa; F2, portal and periportal fibrosis with few septa; F3, portal and periportal fibrosis with numerous septa without cirrhosis; and F4, cirrhosis [13]. A liver biopsy was performed to make a diagnosis for patients with suspected small duct PSC or to exclude other conditions, such as a suspected overlap with autoimmune hepatitis (AIH). The presence of anti-mitochondrial antibodies (AMA) allowed for the exclusion of primary biliary cholangitis (PBC) and an overlap syndrome of PSC and PBC. Patients with PSC were further tested for elevated serum immunoglobulin 4 (IgG4) levels. Among the 74 patients with PSC, 36 (48.6%) had inflammatory bowel diseases (IBD). The laboratory measurements included biochemical tests, mainly of liver enzymes: alkaline phosphatase (ALP), aspartate aminotransferase (AST), alanine aminotransferase (ALT), ɤ-glutamyltransferase (GGT) activities, and total bilirubin concentration. They were elevated and this suggested cholestatic liver disease. The 38 healthy control subjects were recruited from healthy hospital workers (27 females and 11 males, median age: 25 years, range: 21–54). The flow chart of the recruitment and randomization of the patients in the study is presented in Figure 1. Written informed consent was obtained from the patients and healthy subjects after explaining the nature of the study. The study was approved by the local research ethics committee for the Medical University of Bialystok (APK.002.233.2021).

### 2.2. Materials

Our study was retrospective and involved materials collected between January 2015 and December 2015. Findings were obtained immediately or with archival samples. Blood samples were collected from each patient once, after 12 h of fasting in the morning. The sera were separated using centrifugation at 1500× *g* for 10 min, then were frozen and stored at −86 °C until analysis.

### 2.3. Methods

#### 2.3.1. Non-Invasive Laboratory Tests for Liver Fibrosis

The FibroTest (Biopredictive, Paris, France) score was calculated using the following formula [14]:

z = 4.467 log[α2macroglobulin (g/L)] − 1.357 log [haptoglobin (g/L)] + 1.017 log [GGT (IU/L)] + 0.0281 × [age (years)] + 1.737 × [bilirubin (µmol/L)] − 1.184 × [Apo-A1 (g/L)] + 0.301 × gender (female = 0, male = 1) − 5.540.

The score (between 0 and 1) is then:

F = 1/(1 + e^−z^).

The Hepascore (HS) logistic regression model was calculated on the basis of the following formula [15]:

y = exp [−4.185818 − (0.0249 × age) + (0.7464 × gender) + (1.0039 × α2macroglobulin) + (0.0302 × hyaluronic acid) + (0.0691 × bilirubin) − (0.012 × GGT)].

The HS is defined as y/1 + y.

#### 2.3.2. Biochemical Measurements

Biochemical assays, such as the serum ALP (expected range for men is 4–129 IU/L and for women is 35–104 IU/L), AST (expected range: 5–36 IU/L), ALT (expected range for men is 5–41 IU/L and for women is 5–33 IU/L), GGT (expected range for men is 10–71 IU/L and for women is 6–42 IU/L) activity, total bilirubin (expected values for adults are up to 20.52 µmol/L), and total cholesterol (expected values are up to 200 mg/dL) concentrations were measured using routine laboratory methods on a COBAS c501 analyzer (Roche/Hitachi, Tokyo, Japan). The specific proteins of α2-macroglobulin (α2-M), haptoglobin, and apolipoprotein A1 (ApoA1) for formula calculations were determined via the immunoturbidimetric method on a COBAS c501 analyzer (Roche/Hitachi, Tokyo, Japan). The hyaluronic acid (HA) concentration (expected value is 23 ± 17 ng/mL) was also measured via the immunoturbidimetric method with WAKO reagents adapted on the Architect c8000 analyzer (Abbott Laboratories, Abbott Park, Chicago, IL, USA).

#### 2.3.3. Autoantibody Assays

The specific anti-glycoprotein-210 (anti-gp210), anti-sp-100 (anti-sp100), and anti-proteinase 3 antineutrophil cytoplasmic (anti-PR3-ANCA) antibodies were detected using commercially available ELISA kits according to manufacturer’s instructions.

### 2.4. Statistics

The statistical analysis was performed using Statistica 13.3 PL (StatSoft, Kraków, Poland). The normality of distribution was checked using the Kolmogorow-Smirnov test with the Lilliefors correction. This analysis revealed that the distribution of Hepascore does not follow a normal distribution (K-S: d = 0.15811, *p* < 0.05; Lilliefors: *p* < 0.01), but the FibroTest follows a normal distribution (K-S: d = 0.12552, *p* < 0.20: Lilliefors: *p* < 0.01). The differences between the PSC patients and healthy subjects and between the stages of liver fibrosis were evaluated using the Mann–Whitney U test. The effect of the PSC histological changes on the Hepascore and FibroTest was tested using the ANOVA rank Kruskal–Wallis test. The results were expressed as medians and ranges (lower Q1 and upper Q3 quartiles). The correlation between variables was assessed by Spearman’s rank correlation coefficient. The results were considered to be statistically significant when the *p* values were less than 0.05. The diagnostic performance of each test was calculated as the sensitivity, specificity, positive (PPV) and negative predictive values (NPV), and accuracy. To calculate the diagnostic accuracy of the Hepascore and FibroTest, the receiver operating characteristic (ROC) curve was used. GraphROC for Windows (StatSoft, Kraków, Poland) for Windows was used for estimating the clinical characteristics of these tests. For fibrosis and cirrhosis, the target optimal cut-offs were determined according to the Youden method.

## 3. Results

**Figure 2:**
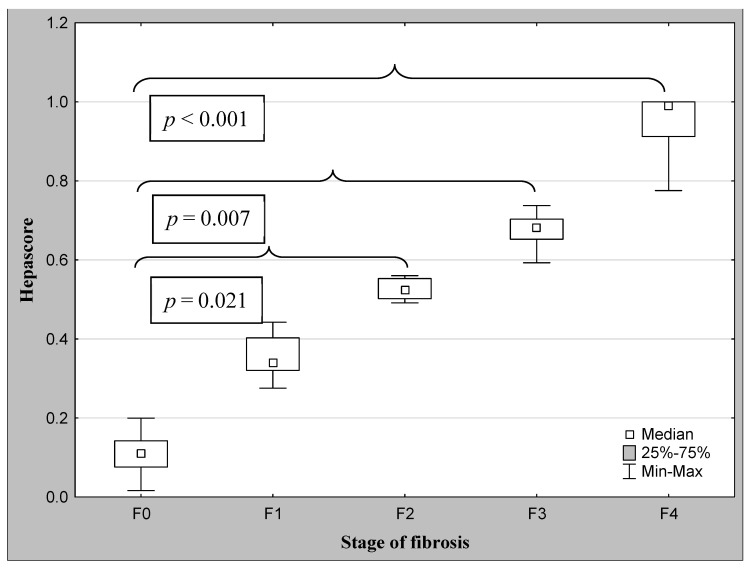
The comparison between the median values of the Hepascore and the stage of fibrosis (post hoc analysis via ANOVA rank Kruskal–Wallis test).

**Figure 3:**
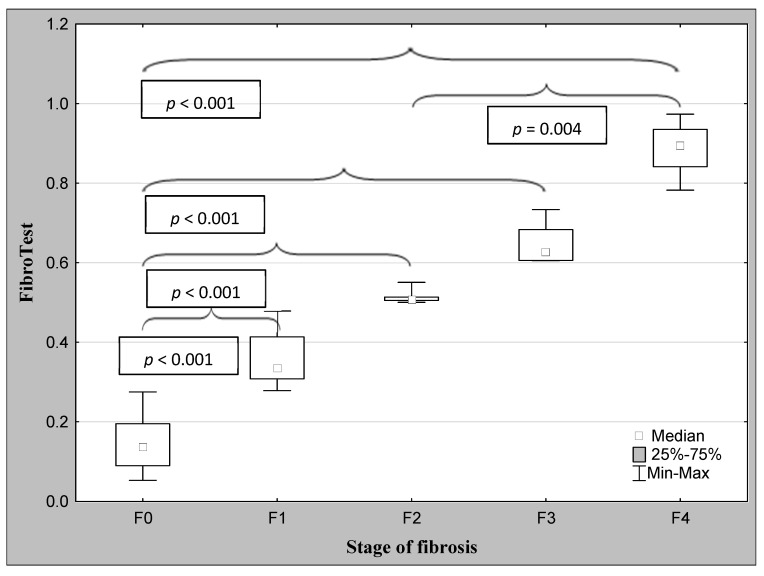
The comparison between the median values of the FibroTest and the stage of fibrosis (post hoc analysis via ANOVA rank Kruskal–Wallis test).

**Figure 4:**
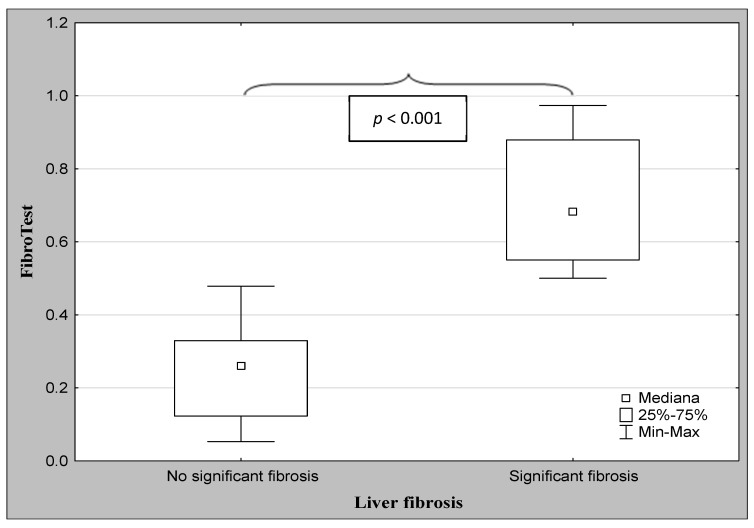
The comparison between the median values of the FibroTest and the severity of liver fibrosis (Mann–Whitney U test).

**Figure 5:**
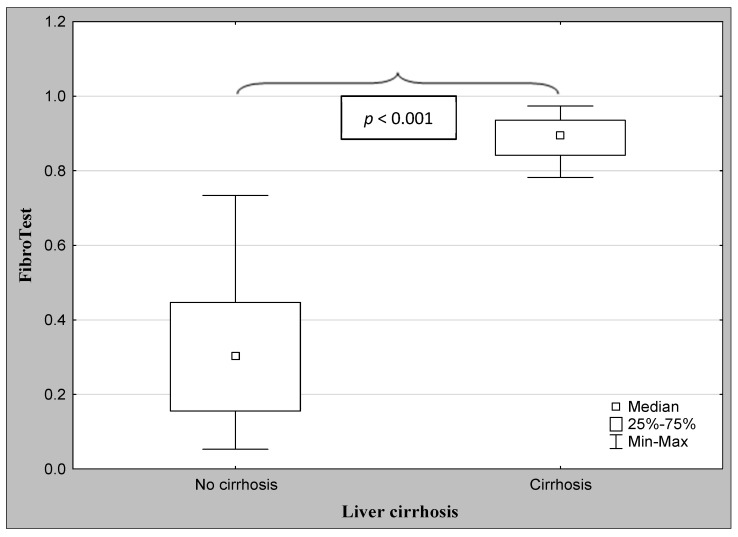
The comparison between the median of the FibroTest and the severity of liver cirrhosis (Mann–Whitney U test).

**Figure 6:**
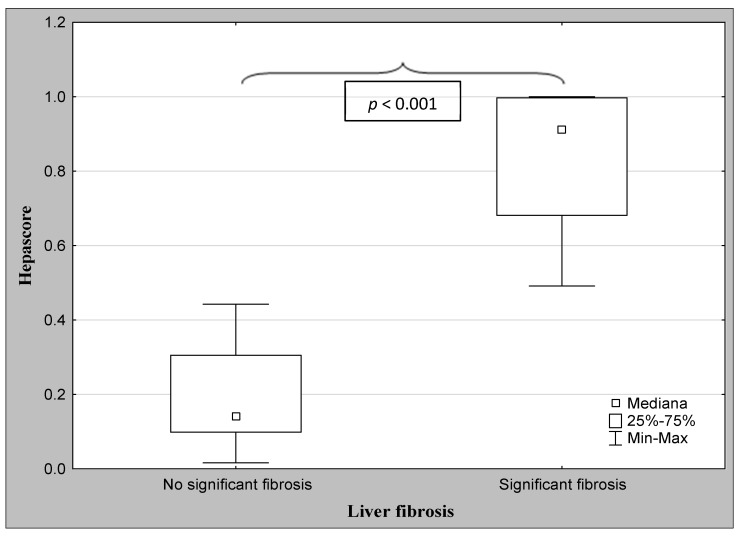
The comparison between the median values of the Hepascore and the severity of liver fibrosis (Mann–Whitney U test).

**Figure 7:**
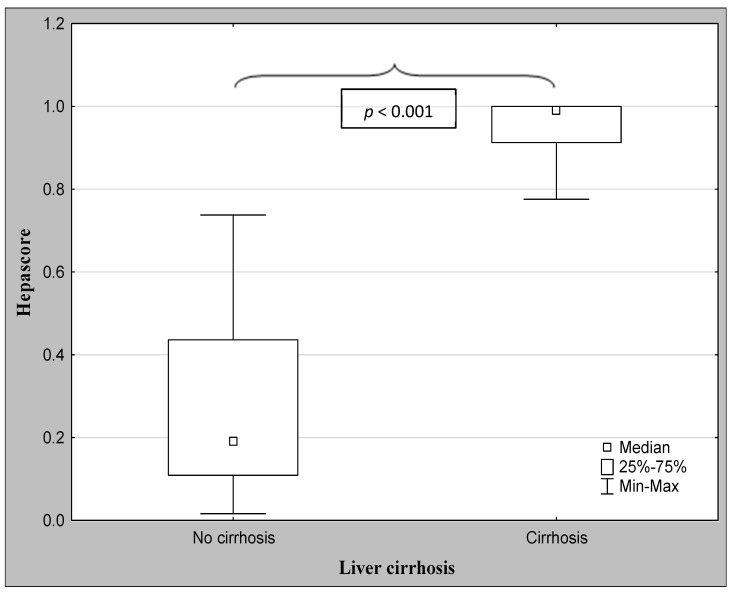
The comparison between the median values of the Hepascore and the severity of liver cirrhosis (Mann–Whitney U test).

**Figure 8:**
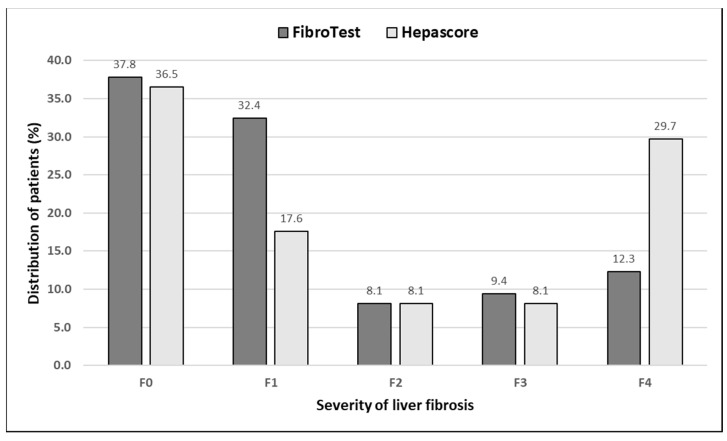
The prevalence (%) of fibrosis and cirrhosis in PSC patients.

The demographic characteristics and biochemical laboratory tests of the PSC patients and healthy subjects are shown in Table 1. The median activity of the serum ALP, GGT, ALT, and α2-macroglobulin concentration were significantly elevated in PSC patients in comparison to the control group. The values of the FibroTest and Hepascore were also significantly higher for those with PSC than those in the controls (*p* < 0.001, *p* = 0.047, respectively). The ANOVA rank Kruskal–Wallis test revealed that there were differences in the Hepascore and FibroTest values between patients divided according to the histological stage as follows: H = 65.93, *p* < 0.001 and H = 66.33, *p* < 0.001. A detailed further post hoc analysis showed that the Hepascore values in patients without fibrosis (F0) were significantly lower than those in stages F2 (*p* = 0.021), F3 (*p* = 0.007), and F4 (*p* < 0.001), although for stage F1, this difference was close to statistically significant (*p* = 0.052). Among patients with different degrees of fibrosis, the values in stage F4 were almost three-fold higher compared to those in stage F1 (Table 2 and Figure 2). Similarly, the values of the FibroTest in stages F1–F3, and F4 were significantly higher than those in the F0 stage (*p* < 0.001 for all comparisons). Among fibrosis, the FibroTest score in F4 was higher than that in the F1 stage (*p* = 0.004) (Table 2 and Figure 3). The Hepascore and FibroTest had significantly higher results in subjects with significant fibrosis (F ≥ 2) in comparison to those with insignificant fibrosis (F1) (*p* < 0.001 for both tests), and higher values in patients with cirrhosis (F4) when compared to those without cirrhosis (F1–F3) (*p* < 0.001 for both comparisons) (Table 3 and Figure 4, Figure 5, Figure 6 and Figure 7). Table 4 presents the values of the biochemical serum markers of the non-invasive tests according to the stages of liver fibrosis in PSC. The ANOVA rank Kruskal–Wallis test showed that there were differences in the HA (H = 16.56, *p* = 0.002), α2-M (H = 16.82, *p* = 0.002), total bilirubin (H = 50.08, *p* < 0.001), and ApoA1 (H = 11.25, *p* = 0.024) between patients, divided according to the histological stage. The values of HA in stages F3 and F4 were higher than those in stage F0 (*p* = 0.045, *p* = 0.006, respectively) (Table 4). This is over a seven-fold increase in stage 4 (cirrhosis) compared to the stage without cirrhosis. The median of the HA concentration was significantly higher in patients with severe fibrosis and cirrhosis compared to those without these changes (*p* < 0.001 for both comparisons) (Table 5). In the case of bilirubin, there were differences between stages F1 and F0 (*p* = 0.013), F2 and F0 (*p* = 0.007), F3 and F0 (*p* < 0.001), F4 and F0 (*p* < 0.001), and between F4 and F1 (*p* = 0.002) (Table 4). In all fibrosis and cirrhosis stages, the concentration of bilirubin was significantly increased. A further post hoc analysis of α2-M showed that its levels in stages F1 and F4 were significantly higher than those in F0 (*p* = 0.009, *p* = 0.022, respectively), whereas the ApoA1 concentrations in stage F4 were lower in comparison to those in F0 and F1 (*p* = 0.022, *p* = 0.015, respectively) (Table 4). The distribution of FibroTest and Hepascore scores is presented in Figure 8. The prevalence of insignificant fibrosis (F < 2) identified via FibroTest was 32.4% (24 of the 74 patients), advanced fibrosis (F ≥ 2)—17.5% (13 patients), and cirrhosis (F4)—12.3% (9 patients). The distribution of the Hepascore identifications was as follows: insignificant fibrosis was 17.6% (13 of the 74 patients), advanced fibrosis—16.2% (12 patients), and cirrhosis—29.7% (22 patients). The diagnostic power of the liver fibrosis markers is presented in Table 6. Considering the Hepascore test, in cut-off point 0.52 in the diagnosis of fibrosis, the sensitivity, specificity, PPV, and NPV were 96.8%, 100%, 100%, and 94.7%, respectively. In point 0.80 for cirrhosis, the sensitivity, specificity, PPV, and NPV were 95.2%, 100%, 100%, and 96.6%. For the other test—the FibroTest, in point 0.51 in the diagnosis of fibrosis, the values were as follows: 58.6%, 90%, 89.5%, and 60%, respectively, and in point 0.73 for cirrhosis: 42.9%, 100%, 100%, and 45.5, respectively. The Hepascore test reached an excellent diagnostic power in identifying both fibrosis and cirrhosis (AUC = 1.000) (Table 6). Spearman’s rank test demonstrated correlations between the FibroTest and Hepascore (R = 0.632, *p* < 0.001), the FibroTest and hyaluronic acid (R = 0.428, *p* < 0.001), and the Hepascore and hyaluronic acid (R = 0.774, *p* < 0.001).

## 4. Discussion

The evaluation of the severity of liver fibrosis is very important for therapeutic strategies, prognosis, and predicting potential complications in patients with primary sclerosing cholangitis. Traditionally, liver biopsy has been considered the gold standard for histologic assessing of the degree of fibrosis (stages) and also the extent of inflammation (grades). This technique is an invasive procedure that can lead to many potential defects and complications. Therefore, a non-invasive assessment of liver fibrosis is needed. Lately, non-invasive techniques, including laboratory tests of liver fibrosis, have been of much interest for the stratification and prognostication of PSC [7,8,9,10,11]. We have to remember that an ideal non-invasive marker for the assessment of fibrosis should be accurate and have high diagnostic sensitivity when detecting the presence of fibrosis, as well as evaluating the stage of liver fibrosis.

We have compared the diagnostic values of two non-invasive, patented markers of liver fibrosis, the FibroTest and Hepascore, in primary sclerosing cholangitis (PSC) cases. To our knowledge, the present study is the first to evaluate these tests as diagnostic and prognostic biomarkers in these patients. In our previous study, we evaluated both the FibroTest and Hepascore in patients with alcoholic liver disease, and also another non-invasive, indirect markers of liver fibrosis in hepatitis C [16,17,18].

As we wrote earlier, a liver biopsy is not necessary for all patients with PSC. When comparing the two techniques, we must be aware of a possible difference in the assessment of the degree of liver failure. In the case of a liver biopsy, it may be due to sampling error or observer error, and in the case of a FibroTest, the cause of false positive errors may be increased values of the algorithm’s components: for clinical reasons (e.g., an increase in bilirubin in Gilbert’s syndrome, or an increase in α2-macroglobulin in inflammation) or from laboratory tests (e.g., an increase in unconjugated bilirubin and a decrease in haptoglobin in hemolysis).

The results of our study showed that the values of both markers differed between the stages of liver fibrosis in primary sclerosing cholangitis. These tests are capable of detecting patients with significant fibrosis (≥F2) and cirrhosis (stage F4). The comparison of the distribution of PSC patients by the severity of liver fibrosis assessed by both markers showed some differences in the number of patients. For instance, the Hepascore appears to be a less sensitive marker in detecting the early histological stage (fewer patients with insignificant fibrosis) and more sensitive in recognizing cirrhosis in comparison to the FibroTest (more than twice as many cirrhotic patients). It seems that some of the patients with minimal and mild fibrosis (F1) remain undiagnosed and classified as persons without fibrosis (F0) (shift to the left). Surprisingly, the Hepascore qualifies more than two times more patients for cirrhosis (stage 4) compared to the FibroTest. It seems that the main confounding factor is a very high hyaluronic acid concentration (algorithm component), which may result in higher Hepascore test values, designating patients into the group with cirrhosis (false-positive results). It is also worth noting that the amount of HA is a good predictor of liver fibrosis, which was also shown in this study [19].

The analysis of the ROC curves shows that the Hepascore and FibroTest can be useful in the diagnosis of liver fibrosis in PSC patients, as AUCs obtained values of 1.000 and above 0.7 (excellent and good diagnostic power, respectively). The Hepascore diagnostic specificity of 100% and PPV of 100% means that there were no false-positive results in the diagnosis of fibrosis and cirrhosis in PSC patients. Similarly, the FibroTest also had a diagnostic specificity and PPV of 100%, but only in the diagnosis of cirrhosis. Comparing the diagnostic sensitivity of both tests, we observed that the sensitivity of the Hepascore for the diagnosis of fibrosis was almost two times higher, and for detecting cirrhosis was more than two times higher than that of the FibroTest. It has been noticed that the most appropriate cut-off point for diagnosing significant fibrosis and mild fibrosis (<F2) and no fibrosis (F0) was 0.52, and for the diagnosis of cirrhosis (F4) or with no cirrhosis (F1–F3) was 0.80. For comparison, in our previous study with alcoholic patients, the cut-off point of Hepascore for differentiation between stages F0–F2 and ≥F2 was only 0.20 [17]. In the study by Adams et al., Hepascore was validated as a predictor of liver fibrosis in chronic hepatitis C infection [20]. These authors have shown that at a score equal to or higher than 0.50 (a cut-off value similar to the one in our study), the diagnostic specificity and sensitivity were 81% and 95% for advanced fibrosis, and at the score below 0.84 (cut-off point similar to the one in our study), they were 84% and 71%, respectively, for cirrhosis. The cut-off points of the FibroTest indicated by the ROC curve for the differentiation between insignificant fibrosis and significant fibrosis and no cirrhosis and cirrhosis were 0.51 and 0.73. In our previous study performed with alcoholic patients, the cut-off point for the differentiation between stages F0–F2 and F3–F4 was lower and obtained the value of 0.21 with a diagnostic specificity of 93.9% and sensitivity of 61.3% (18). In turn, in the overview of the diagnostic value of biochemical markers of liver fibrosis in the integrated database of 1570 patients with hepatitis C, at a cut-off of 0.31, the negative predictive value for excluding significant fibrosis was 91% with a diagnostic specificity of 68%, a diagnostic sensitivity of 84%, and for the diagnosis of significant fibrosis via the METAVIR scoring system, the AUROCs ranged from 0.73 to 0.87 [21]. In another study, the standardization of the ROC curve for the diagnostic evaluation of liver fibrosis markers based on the prevalence of the fibrosis stage showed, considering stage prevalence, that the FibroTest AUC for advanced fibrosis varied from 0.67 (only stage F2 as advanced fibrosis and only F1 as nonadvanced fibrosis) to 0.98 (only F4 as advanced fibrosis and only F0 as nonadvanced fibrosis), and considering the fibrosis stage prevalence—the FibroTest AUC varied from 0.65 to 0.89 [22].

The limitation of this study is the small sample size of the subgroups selected according to histological stages. Therefore, further studies are needed.

## 5. Conclusions

In summary, we concluded that both the patented, non-invasive markers, FibroTest and Hepascore, are highly valuable for the diagnosis of the severity of liver fibrosis and cirrhosis in patients with primary sclerosing cholangitis (PSC) and can be used as a primary screening method instead of a liver biopsy. Both markers have the required sensitivity and specificity to detect liver fibrosis and cirrhosis and can be equally used in clinical practice, although the Hepascore seems to be a better test because it is more specific.

## Figures and Tables

**Table 1:** Demographic and biochemical characteristics of the study population.

	PSC (*n* = 74)	Healthy Subjects (*n* = 38)	*p*-Value
Age (years) (median/range)	36 (26–75)	25 (21–54)	-
Sex (Female/Male)	27/47	27/11	-
ALP (IU/L)	190 91.0–331.0	59.0 53.0–70.0	*p* < 0.001 *
GGT (IU/L)	255.7 111.5–532.2	23.5 18.5–28.0	*p* < 0.001 *
AST (IU/L)	24.9 14.4–48.7	23.0 19.0–26.0	*p* = 0.541
ALT (IU/L)	34.4 21.8–67.0	17.5 11.5–19.5	*p* < 0.001 *
Bilirubin (µmol/L)	7.69 6.16–15.05	13.42 7.27–17.1	*p* = 0.222
α2-macroglobulin (g/L)	2.33 1.80–2.82	1.84 1.47–2.18	*p* < 0.001 *
Haptoglobin (g/L)	0.99 0.61–1.48	0.98 0.77–1.21	*p* = 0.684
ApoA1 (g/L)	1.60 1.28–1.91	1.52 1.39–1.72	*p* = 0.455
FibroTest	0.32 0.17–0.51	0.10 0.07–0.15	*p* < 0.001
Hepascore	0.39 0.14–0.82	0.27 0.21–0.27	*p* < 0.047
Hyaluronic acid (ng/mL)	40.8 18.5–95.0	35.0 25.0–40.5	*p* = 0.259
Anti-PR3-ANCA (+/−) (%)	25/49 (34.3)	ND	-
Anti-sp100 (+/−) (%)	2/72 (2.94)	ND	-
Anti-gp210 (+/−) (%)	4/70 (4.90)	ND	-

Data are presented as medians and quartiles (Q1 and Q3). The differences between PSC and healthy subjects group were estimated via Mann–Whitney U test. * Significant difference at *p* < 0.05. Abbreviations: PSC, primary sclerosing cholangitis; ALP, alkaline phosphatase; GGT, γ-glutamyltransferase; AST, aspartate aminotransferase; ALT, alanine aminotransferase; ApoA1, apolipoprotein A1; Anti-PR3-ANCA, anti-proteinase 3 antineutrophil cytoplasmic antibody; Anti-sp-100, anti-sp-100 antibodies; Anti-gp-210, anti-glycoprotein-210 antibodies; ND, not detectable.

**Table 2:** The values of non-invasive markers according to stage of liver fibrosis in PSC.

Marker	F0	F1	F2	F3	F4
FibroTest	0.14 0.09–0.19 (*n* = 28)	0.33 0.31–0.41 (*n* = 24)	0.51 0.50–0.51 (*n* = 6)	0.63 0.60–0.68 (*n* = 7)	0.89 0.84–0.93 (*n* = 9)
Hepascore	0.11 0.08–0.14 (*n* = 27)	0.34 0.32–0.40 (*n* = 13)	0.52 0.50–0.55 (*n* = 6)	0.68 0.65–0.70 (*n* = 5)	0.99 0.91–1.00 (*n* = 22)

Data are presented as medians and quartiles (Q1 and Q3). F0–F4, histology scored according to the Ludwig’s score system. The values of the FibroTest and Hepascore differed between the stages of liver fibrosis via the ANOVA rank Kruskal–Wallis test. A further post hoc analysis is presented in the Results section and Figure 1 and Figure 2.

**Table 3:** The value of non-invasive markers in patients with significant fibrosis, no significant fibrosis, without and with cirrhosis.

Marker	No Significant Fibrosis (F1)	Significant Fibrosis (F ≥ 2)	Without Cirrhosis (F1–F3)	Cirrhosis (F4)
Hepascore	0.14 0.10–0.30 *n* = 40	0.91 0.68–1.00 *n* = 33	0.18 0.11–0.42 *n* = 51	0.99 0.87–1.00 *n* = 23
		*p* < 0.001 *		*p* < 0.001 ^#^
FibroTest	0.26 0.12–0.33 *n* = 52	0.68 0.55–0.88 *n* = 22	0.30 0.15–0.44 *n* = 64	0.89 0.78–0.93 *n* = 10
		*p* < 0.001 *		*p* < 0.001 ^#^

Data are presented as medians and quartiles (Q1 and Q3). The differences between significant fibrosis and no significant fibrosis (*p* *), and cirrhosis and without cirrhosis (*p* ^#^) are estimated via Mann–Whitney U test. Significant difference at *p* < 0.05.

**Table 4:** The value of biochemical serum markers of non-invasive tests according to stage of liver fibrosis in PSC.

Marker	F0	F1	F2	F3	F4
HA	24.7 7.65–40.0 (*n* = 28)	35.0 21.1–88.9 (*n* = 24)	33.8 17.9–145 (*n* = 6)	95.0 43.4–142 (*n* = 7)	178.5 96.4–224 (*n* = 9)
α2-M	1.77 1.54–2.36 (*n* = 28)	2.54 2.03–3.01 (*n* = 24)	2.43 2.01–2.95 (*n* = 6)	2.47 2.22–2.80 (*n* = 7)	2.70 2.33–3.22 (*n* = 9)
GGT	165 53.4–353 (*n* = 14)	257.5 111.5–557 (*n* = 9)	545 545–545 (*n* = 1)	298 263–557 (*n* = 4)	429 242–462 (*n* = 4)
Bilirubin	6.16 4.45–6.16 (*n* = 28)	8.55 6.33–11.2 (*n* = 24)	14.9 7.69–15.9 (*n* = 6)	22.4 11.6–25.8 (*n* = 7)	46.2 40.5–60.7 (*n* = 9)
Hp	0.99 0.67–1.67 (*n* = 28)	0.81 0.52–1.12 (*n* = 24)	0.62 0.45–1.24 (*n* = 6)	0.91 0.21–1.24 (*n* = 7)	1.11 0.26–1.74 (*n* = 9)
ApoA1	1.70 1.44–2.01 (*n* = 28)	1.73 1.52–1.91 (*n* = 24)	1.69 1.42–1.84 (*n* = 6)	1.70 1.30–2.20 (*n* = 7)	0.58 0.31–1.16 (*n* = 9)

Data are presented as medians and quartiles (Q1 and Q3). F0–F4, histology scored according to the Ludwig’s score system. The values of markers differed between the stages of liver fibrosis via ANOVA rank Kruskal–Wallis test. A further post hoc analysis is presented in the Results section. Abbreviations: HA, hyaluronic acid; α2-M, α2-macroglobulin; GGT, γ-glutamyltransferase, Hp, haptoglobin; ApoA1, apolipoprotein A1.

**Table 5:** The values of hyaluronic acid in patients with significant and no significant fibrosis, and without and with cirrhosis.

Marker	No Significant Fibrosis (F1) (*n* = 52)	Significant Fibrosis (F ≥ 2) (*n* = 22)	Without Cirrhosis (F1–F3) (*n* = 64)	Cirrhosis (F4) (*n* = 10)
Hyaluronic acid	28.9 15.6–52.1	95.8 28.1–219	34.0 16.9–66.5	167 69.2–224
		*p* < 0.001 *		*p* < 0.001 ^#^

Data are presented as medians and quartiles (Q1 and Q3). The differences between significant fibrosis and no significant fibrosis (*p* *), and cirrhosis and without cirrhosis (*p* ^#^) were estimated via Mann–Whitney U test. Significant difference at *p* < 0.05.

**Table 6:** The diagnostic power of liver fibrosis markers in PSC.

Marker	Cut-Off	Sensitivity (%)	Specificity (%)	ACC (%)	PPV (%)	NPV (%)	AUC ± SE
Hepascore							
-for fibrosis	0.52 *	96.8	100	98.0	100	94.7	1.000 ± 0.00
-for cirrhosis	0.80 ^#^	95.2	100	98.0	100	96.6	1.000 ± 0.00
FibroTest							
-for fibrosis	0.51 *	58.6	90.0	71.4	89.5	60.0	0.769 ± 0.07
-for cirrhosis	0.73 ^#^	42.9	100	61.3	100	45.5	0.767 ± 0.09

* cut-off point < F2. ^#^ cut-off point < F4. To calculate the diagnostic accuracy of Hepascore and FibroTest, GraphROC for Windows was used. For fibrosis and cirrhosis, target optimal cut-offs were determined according to the Youden method. Abbreviations: PSC, primary sclerosing cholangitis; ACC, diagnostic accuracy; PPV, positive predictive value; NPV, negative predictive value; AUC, area under ROC curve; SE, standard error.

## Data Availability

No new data were created or analyzed in this study. Data sharing is not applicable to this article.
